# Optimizing the Use of Computed Tomography for Appendicitis Diagnosis in the Pediatric Emergency Department Through the Quality Improvement Methodology

**DOI:** 10.7759/cureus.75760

**Published:** 2024-12-15

**Authors:** Ahmed Elhatw, Jonathan E Teitelbaum, Ojas Chinchwadkar

**Affiliations:** 1 Pediatrics, Monmouth Medical Center, Long Branch, USA; 2 Pediatric Gastroenterology, Monmouth Medical Center, Long Branch, USA; 3 Pediatrics, Rutgers Robert Wood Johnson Medical School, New Brunswick, USA

**Keywords:** abdominal ct scan, alvarado score, pediatric appendicitis, radiation exposure, right lower quadrant ultrasound

## Abstract

Background: Acute appendicitis is one of the most common causes of an acute abdomen among pediatric patients. The diagnosis of appendicitis is challenging due to the nonspecific presentation. Diagnosis is based on historical, physical, and serologic information as well as right lower quadrant ultrasound (RLQ US). In equivocal patients, or those with a high degree of suspicion, computed tomography (CT) of the abdomen and pelvis with intravenous (IV) contrast can be utilized to rule in appendicitis. However, optimizing diagnostic protocols to minimize ionizing radiation exposure while maintaining diagnostic accuracy is important.

Methods: We performed a monthly retrospective analysis of CT usage among pediatric patients with suspected acute appendicitis presenting to the pediatric emergency department (ED) from June 2023 to December 2023. We used quality improvement methodology to decrease CT use with monthly Plan-Do-Study-Act (PDSA) cycles with an aim to decrease CT use by 50%. The main intervention was coordination between the ED and surgical providers to require surgical consult before ordering a CT. We quantified the number of patients who received a surgical consult before CT, the number of RLQ US performed, the number of CTs performed, and the number of appendectomies, specifically the number with perforation.

Results: A total of 249 patients under 18 years of age presented to the pediatric ED with symptoms of acute appendicitis during the study period. All 249 patients underwent an initial RLQ US. The number of CTs performed decreased from a baseline of 14 in June to a nadir of four in September (71% decrease, p=0.029). There was a decrease in the percentage of patients who underwent a CT scan after an RLQ US from 36.5% in June to 11.8% in September after our intervention. In June, a total of 38 RLQ US were performed and 14 patients underwent additional CT (36.5%) and in September a total of 34 US were performed and 14 patients underwent additional CT (11.8%). There was an increase in the surgical consults rate from a baseline of seven surgical consults with 14 total CTs in June 2023 (50%) to seven consults with a total of seven CTs performed (100%) in December 2023. There was no increase in appendiceal perforation rates.

Conclusion: Multidisciplinary discussions between pediatric ED physicians and pediatric surgeons reduced CT usage, and corresponding radiation exposure and cost, in the evaluation of appendicitis.

## Introduction

Acute appendicitis is the most common cause of the acute abdomen, with an incidence of 1 per 1000 persons per year [1,2]. Diagnosis of acute appendicitis is dependent on clinical examination, laboratory studies, and ultrasound (US) findings. In some instances, performing computed tomography (CT) of the abdomen and pelvis with intravenous (IV) contrast could help establish the diagnosis. The presentation of acute appendicitis can be nonspecific, leading to delays in diagnosis and higher rates of perforation [3]. A retrospective study in 2015 revealed there is no significant difference in pathology-proven appendicitis, perforation, or negative appendectomy among patients who underwent right lower quadrant US (RLQ US) versus CT evaluation [4].

Exposure to radiation poses many health hazards to pediatric patients; hence, the decision to perform a CT scan must weigh the risks and benefits and should include sufficient clinical indications. CT scans increase the risk of developing childhood cancers such as brain tumors and bone marrow malignancies such as leukemia. There is a positive correlation between the dosage of radiation exposure and the likelihood of developing childhood cancer [5,6].

Our aim was to use quality improvement (QI) methodology to decrease the use of CT in evaluating pediatric appendicitis in our emergency department (ED) by 50% over a period of six months.

## Materials and methods

Design and setting

This study is a retrospective observational QI study performed in the pediatric ED at a community teaching hospital.

Selection of participants

Using the electronic health care software system “Epic” slicer/dicer feature (Epic Systems Corporation, Verona, USA), we identified all patients less than 18 years of age who underwent CT to rule out appendicitis in the pediatric ED at our hospital from June 2023 to December 2023. We excluded patients over the age of 18, patients in which CT scans were performed to rule out diagnoses such as renal stones, and post-appendectomy CT scans that were performed due to suspicion of postoperative complications.

Methods

After Institutional Review Board approval, monthly data was retrospectively obtained from the period of June 2023 to December 2023 and included patient age, sex, US result, CT result, pathological diagnosis, and whether the surgical team was consulted before performing the CT scan. Data was retrospectively analyzed at the end of each month. We performed chart reviews for each patient and evaluated both US and CT results. We determined if the US was performed before the CT scan and if the surgical consult was placed in advance before performing the CT scan. Baseline data was obtained in June 2023. These and subsequent monthly results were shared with the pediatric ED staff and the surgical team through December 2023.

Statistics

A simple 2x2 chi-square contingency calculator was used to determine if the number of CT scans performed each month versus “not performed” was different from the baseline obtained in June. There was 1 degree of freedom. The chi-square statistic with Yates correction was determined. Significance was set at a p-value less than 0.05.

Intervention

The initial intervention in July 2023 was a change in the clinical workflow ensuring that a surgical consult was completed before ordering a CT. This workflow plan was discussed and reinforced monthly with the pediatric surgery attendings, pediatric emergency physicians, and physician assistants covering the pediatric ED as part of each Plan-Do-Study-Act (PDSA) cycle. Thus, an abdominal CT was only ordered if the ED clinician and the surgical team were in agreement and after the US report was available and discussed with the radiology department.

## Results

A total of 55 CTs and 249 RLQ US were performed during the study period from June 2023 to December 2023. All patients underwent RLQ US before performing CT scans. Among patients who underwent CT scans, about half were males (51%), and the average age was 11.3 years (Table 1). The various CT diagnoses are found in Table 1 with acute appendicitis being the most frequent (36.3%).

**Table 1:** Demographic data of patients who underwent a CT scan

Characteristics	Total (n=55)
Gender	
Male	28 (51%)
Female	27 (49%)
Age	
Median	11.3 years
Range	4-17 years
CT diagnosis	
Appendicitis	20 (36.3%)
Normal/non-specific findings	18 (32.7%)
Lower lobe pneumonia	1 (1.8%)
Colonic wall thickening	5 (9%)
Adnexal mass	3 (5.4%)
Cystitis	1 (1.8%)
Mesenteric adenitis	4 (7.27%)
Renal stone	3 (5.45%)

Table 2 reveals the total number of CTs and US performed each month both at baseline, in June, and post-intervention. In June, of the 38 patients receiving RLQ US for suspected appendicitis, 14 received a CT (36.5%). In September, of 34 patients receiving RLQ US, only four received a CT (11.8%). The decrease in CTs for the six months post-intervention was statistically significant (χ^2^(1, 249)=4.71; p=0.030).

**Table 2:** Total number of CT scans and ultrasounds performed per month

Month	Total number of CT scans	Total number of ultrasound	Percentage of total patients who underwent CT scan (%)	χ² with Yates correction (p-value)
June	14	38	36.8	
Intervention				
July	11	36	30.5	0.11 (0.745)
August	9	36	25	0.72 (0.396)
September	4	34	11.8	4.76 (0.029)
October	5	27	18.5	1.75 (0.186)
November	5	28	17.8	1.98 (0.159)
December	7	50	14	5.01 (0.025)

**Figure 1:**
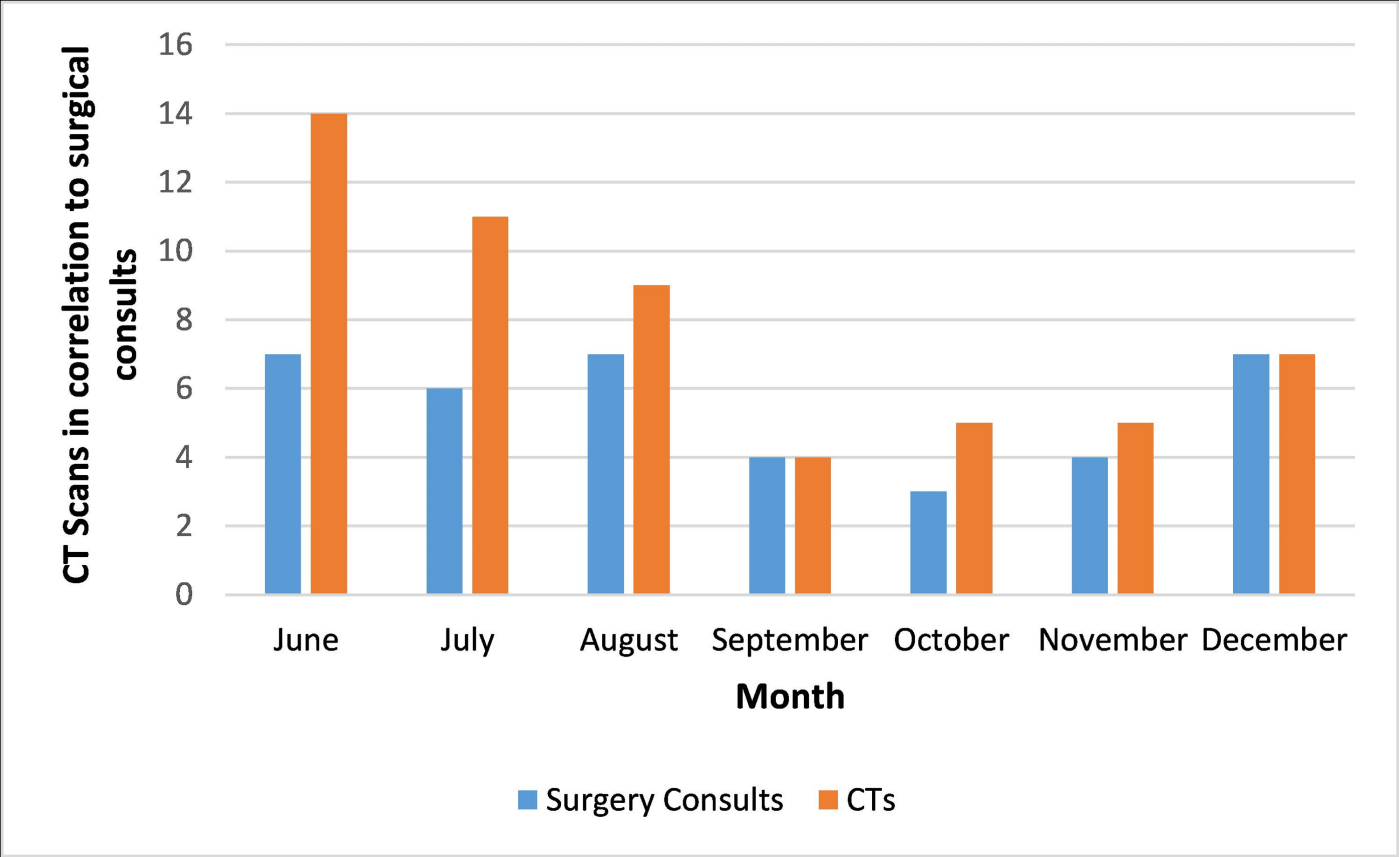
Number of CTs and surgery consults prior to CT by month

Surgical consults were obtained before seven out of the 14 CTs ordered in June (50%). After our intervention, they were obtained before 38 out of the 41 CTs ordered in July through August (92.6%) (Figure 1).

**Figure 2:**
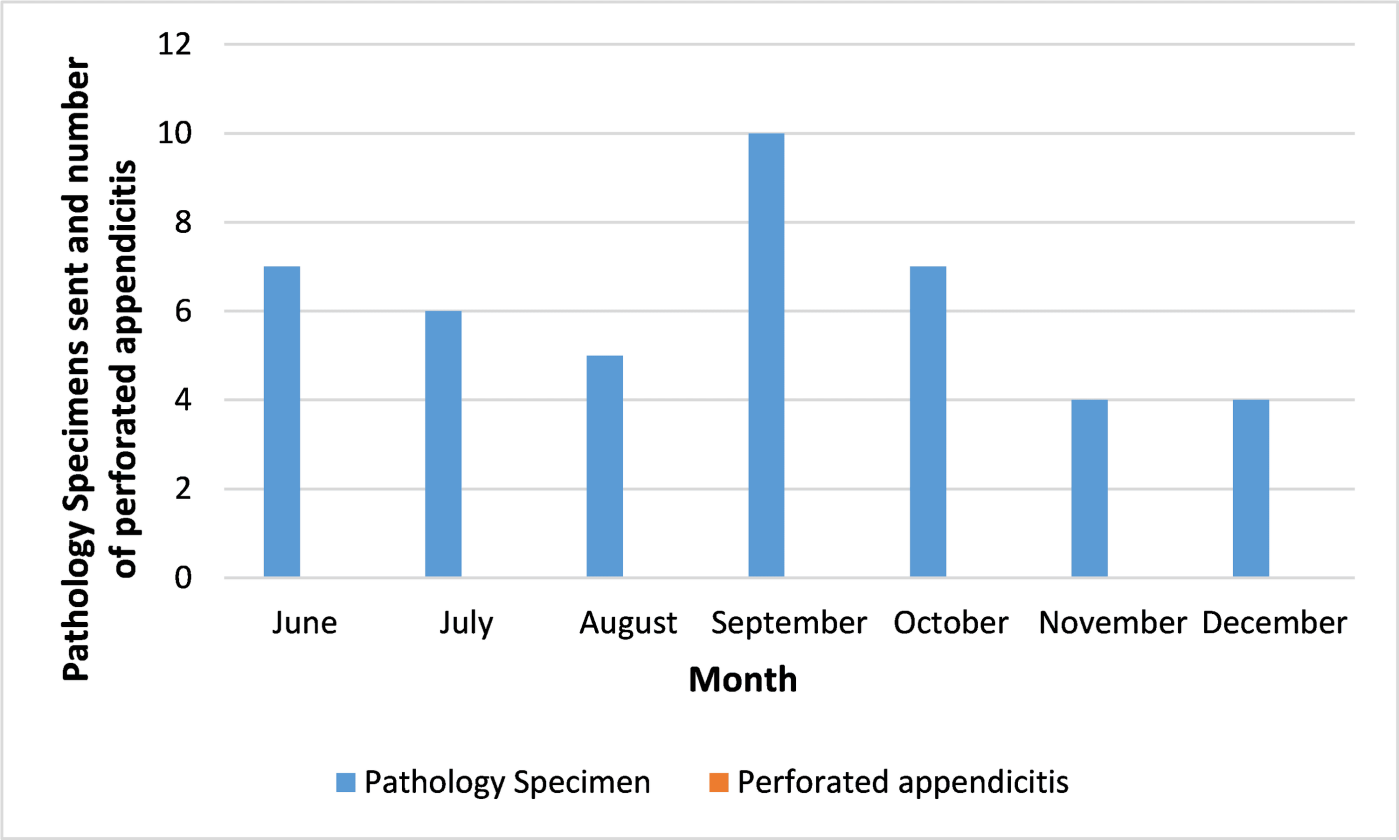
Number of specimens sent to pathology per month There was no perforated appendicitis.

Evaluation of the surgical pathology specimens (n=43) during the entire study period revealed there were no perforated appendices (Figure 2).

## Discussion

The evaluation of patients with suspected appendicitis initially includes a thorough history, physical exam, and interpretation of laboratory tests. Based on this, the clinician must decide if further imaging is warranted. US is the best initial investigation for evaluating appendicitis as it does not involve radiation. However, the localization of the appendix is challenging due to anatomical variations, and failure to identify the segment or tip can lead to an inaccurate diagnosis [3,7]. Even if the appendix is not visualized, secondary US findings such as free fluid and fat stranding, in correlation with laboratory and clinical findings, can be sufficient for the diagnosis of appendicitis without the need to perform a CT [8]. Darbyshire et al. performed a retrospective cohort study evaluating children aged 5 to 16 years who were admitted to the surgical unit for appendectomy. US showed a sensitivity (0.9, 95% confidence interval (CI): 0.86-0.96) and specificity (1.0, 95% CI: 0.96-1.0). The rate of negative appendectomy was low at 1.4% [9].

The Pediatric Surgery Quality Collaborative performed a national study to compare the use of CT utilization among 42 children’s hospitals in the United States. Structured interviews were performed with surgeons, radiologists, and ED physicians and their results showed that hospitals with low CT use rates had consistent available resources, adherence to protocols guiding imaging modality, and a culture of interdepartmental collaboration [10]. A study by the National Hospital Ambulatory Medical Care Survey showed an increase in CT usage to rule out appendicitis in pediatric patients from 0.9% in 1998 to 15.4% in 2008 (p<0.001) [11]. A 2013 study noted the odds of performing CT scans were significantly lower in pediatric centers that use US as a first imaging modality [12]. Many institutes perform CT only with equivocal findings or with suspicion of complications in order to reduce the risk of radiation [5,6,13]. Saito et al. in 2013 retrospectively reviewed children who underwent appendectomies at a tertiary pediatric hospital. Most children (395/423, 93.4%) had preoperative imaging. Multivariate analysis showed that initial evaluation at a community hospital was associated with 4.4 higher odds of obtaining a preoperative CT scan (p=0.002) compared to preoperative US (odds ratio: 0.20; p=0.003) [14].

In our study, QI methodology was used to successfully decrease the use of CT in the evaluation of children with suspected appendicitis. During the pre-intervention month of June 2023, all 38 children presenting to the ED with concerns for appendicitis were evaluated and underwent RLQ US, and 14 subsequently underwent CT. The decision as to whether to proceed to CT was made only by the ED physician, without the input of the surgical team, in seven of the 14 patients (50%). The intervention implemented by the authors of the study was to request a surgical consult prior to obtaining a CT. The authors hypothesized that involving the surgical team in the decision of whether or not to obtain a CT would lead to decreased CT usage. This sole intervention led to a dramatic increase to 100% in obtaining a surgical consult prior to the CT by the end of the study period. There was a corresponding significant decrease (p=0.030) in CT use during the subsequent six months of data collection. By reviewing pathology records, there were no perforated appendix during this study period, suggesting that this decrease in CT use did not result in patient harm. Further, the consistently stable number of RLQ US during each month of the study period suggests that the decrease in CT use was not secondary to a decrease in patients presenting to the ED with concerns of appendicitis. This finding is supported by the decrease in the percentage of patients who had a CT each month during the study period from a baseline of 36.8% to a nadir of 11.8%. Our practice is in alignment with recommendations from the American College of Radiology criteria to perform a US before a CT scan in evaluating appendicitis [15]. Our practice is also aligned with the American Academy of Pediatrics (AAP) policy statement to optimize the use of advanced imaging by having clinical decision-making support mechanisms before ordering CT scans [16].

Gurien et al. performed a retrospective observational study and decreased CT usage from 94.2% to 27.5% (p<0.001) through the use of an appendicitis diagnosis pathway. This pathway ensured that patients get a US before CT scan with IV contrast, a practice that already existed in our institution. Our study was designed to further decrease the percentage by including the surgical team in the workflow [17]. AlFraih et al. in 2017-2018 similarly attempted to decrease CT use by using QI methodology and a multidisciplinary approach, which included implementing the use of the pediatric appendicitis score, standard US reporting, and risk stratification. The rate of CT usage decreased from 7.3% to 4.7% and negative appendectomy decreased from 4% to 1.5% after pathway implementation [18]. Similar to our study, in a study performed by Alter et al., CT was only obtained if requested by the consulting pediatric surgeon. Their results showed follow-up CT scans were obtained in 19% of all patients, including 8% with positive US, 4% negative, 32% borderline, and 22% when the appendix was not visualized [19].

Limitations

The decision to perform a CT is dependent on shared decision-making between the patient, their family, the surgeon, and the ED clinician. This decision is somewhat subjective and clinician-dependent. While we saw no incidence of perforated appendicitis during the study period, it is possible that patients presented to a different ED with complicated appendicitis after a misdiagnosis in our institution.

## Conclusions

Based on our study, multidisciplinary discussions between pediatric ED physicians and pediatric surgeons can reduce CT usage, and corresponding radiation exposure, in the evaluation of appendicitis. Our multidisciplinary approach did not result in an increased risk of perforated appendicitis on pathological analysis. We recommend the performance of CT scans only in equivocal cases in which the US does not give conclusive findings and only when the ED provider and surgical team are in agreement. Our multidisciplinary approach will help reduce the risk of radiation exposure in patients who present to pediatric ED for evaluation of appendicitis in addition to reducing the healthcare costs.
